# Decreased brain-derived neurotrophic factor plasma levels in psoriasis patients

**DOI:** 10.1590/1414-431X20154574

**Published:** 2015-07-21

**Authors:** A.R. Brunoni, P.A. Lotufo, C. Sabbag, A.C. Goulart, I.S. Santos, I.M. Benseñor

**Affiliations:** 1 Centro de Pesquisa Clínica e Epidemiológica, Hospital das Clínicas, Universidade de São Paulo, São Paulo, SP, Brasil; 2 Faculdade de Medicina, Universidade de São Paulo, São Paulo, SP, Brasil

**Keywords:** Brain derived neurotrophic factor, Psoriasis, Neurotrophins, Psychological stress, Case-control study

## Abstract

Brain-derived neurotrophic factor (BDNF) is associated with neuroplasticity and synaptic strength, and is decreased in conditions associated with chronic stress. Nevertheless, BDNF has not yet been investigated in psoriasis, a chronic inflammatory systemic disease that is exacerbated by stress. Therefore, our aim was to determine BDNF plasma levels in psoriasis patients and healthy controls. Adult patients (n=94) presenting with psoriasis for at least 1 year were enrolled, and age- and gender-matched with healthy controls (n=307) from the Brazilian Longitudinal Study of Adult Health (ELSA-Brasil). Participants had neither a previous history of coronary artery disease nor current episode of major depression. BDNF plasma levels were determined using the Promega ELISA kit. A general linear model was used to compare BDNF levels in psoriasis patients and controls, with age, gender, systolic blood pressure, serum fasting glucose, blood lipid levels, triglycerides, smoking status, and body mass index examined. After adjusting for clinical and demographic variables, significantly decreased BNDF plasma levels were observed in psoriasis patients (P=0.01) (estimated marginal means of 3922 pg/mL; 95%CI=2660-5135) compared with controls (5788 pg/mL; 95%CI=5185-6442). Similar BDNF levels were found in both mild and severe cases of psoriasis. Our finding, that BDNF is decreased in psoriasis, supports the concept of a brain-skin connection in psoriasis. Further studies should determine if BDNF is increased after specific psoriasis treatments, and associated with different disease stages.

## Introduction

Psoriasis is a chronic inflammatory disease associated with stress and increased prevalence of mental disorders (1). Recent studies have highlighted the role of neuropeptides (2) such as neurotensin (3) in psoriasis, possibly because of mast cell activation predisposing towards skin inflammation and the release of other neuroinflammatory cytokines (4). Brain-derived neurotrophic factor (BDNF) is a neurotrophin associated with neuroplasticity, neuronal growth, and synaptic strength (5), and is involved in functions such as memory and learning (5). Additionally, BDNF is decreased in depression (6). Indeed, animal studies show that experimentally induced stress reduces BDNF transcription and synthesis (7,8). Thus, we aimed to determine if BDNF plasma levels are decreased in psoriasis patients compared with controls.

## Material and Methods

### Subjects

This is an ancillary study of CALIPSO (Coronary Artery Calcium in Psoriasis), a cross-sectional study investigating psoriasis, as described elsewhere (9,10), and the Brazilian Longitudinal Study of Adult Health (ELSA-Brasil), a large cohort study of 15,105 Brazilian civil servants, again described elsewhere (11). The period of data collection was from January to December 2012. The local and national Ethics Committee approved the study protocol and participants provided informed, written consent.

The cases (n=94) were adult patients (>35 and 40 years for men and women, respectively) presenting with psoriasis for at least 3 years prior to entering the study, as diagnosed by an expert dermatologist in a referral outpatient clinic. Mild cases were defined by the absence of psoriatic arthritis and use of a systemic treatment (e.g., methotrexate, cyclosporine, mycophenolate mofetil, immunobiological treatments, or phototherapy). Severe cases fulfilled at least one of these conditions. Patients were excluded if they had coronary artery disease or depression (diagnosed by scores ≥10 in the Patient Health Questionnaire-9) (12).

The control group (n=307) was composed of age- and sex-matched participants from ELSA-Brasil (11). Subjects were only selected from the São Paulo study center (5061 participants) to ensure that both the cases and controls were from the same study site. Potential controls were excluded if they had coronary artery disease or a current depressive episode, according to the Portuguese adapted version of the Clinical Interview Schedule Revised, CIS-R (13). Each case was then matched at random to three controls of the same age and gender.

### Plasma BDNF levels

Blood samples were obtained from the antecubital vein into EDTA-treated tubes. Samples were spun at 3000 g for 15 min at 5°C, and the collected plasma stored at -80°C until analysis. An enzyme-linked immunosorbent assay (ELISA) kit (Promega, Switzerland) and microplate reader (at 450 nm) were used to determine BDNF values. Mean intra-assay and inter-assay coefficients of variation were 4.1 and 5.4%, respectively. The assay sensitivity threshold was 2 pg/mL. Sample concentrations in each plate were calculated from standard curves and dilution factors.

### Data collection

All subjects completed an extensive survey regarding previous and family history of diabetes, hypertension, dyslipidemia, angina, coronary heart disease, myocardial infarction, revascularization (percutaneous or surgery), education, and smoking. Anthropometric parameters were measured using standard equipment and techniques (Anthropometry Procedures Manual, Center for Disease Control, 2004). Blood samples were collected after a 12-h overnight fast. Fasting glucose was estimated by the hexokinase method. Total cholesterol, high-density lipoprotein cholesterol (HDLc), and triglycerides were determined by enzymatic colorimetric assays, with glycerol phosphate peroxidase used for triglycerides. Low-density lipoprotein cholesterol (LDLc) was calculated from the Friedewald equation, except in participants with triglycerides >400 mg/dL, for whom LDLc was estimated using an enzymatic colorimetric assay.

### Statistical analysis

All statistical analyses were performed using Stata 12 (Statacorp, USA). The sample size needed to detect a difference in BDNF levels corresponding to, at least, a small-to-medium effect size (Cohen’s *d*=0.3 or sample size difference corresponding to 0.3 SD of the total sample) was estimated. Based on our meta-analysis of BDNF in depression (6), the mean/SD ratio was estimated to be 1. A 1:1 sample size ratio (i.e., 94 cases and 94 controls) was initially considered, but this yielded a statistical power of only 0.69. Additional power analyses were performed by increasing the number of controls by two- and three-fold, obtaining statistical powers of 0.76 and 0.83, respectively. Importantly, the number of cases could not be increased, as blood samples were not available. Therefore, to increase the study power, we chose to remove the matching and perform an unconditional analysis.

The variables were normally distributed; therefore Student’s *t*-tests and the chi-square test were used to compare clinical and demographic data between groups. Measured variables included age, gender, systolic blood pressure (SBP), serum fasting glucose, HDLc, LDLc, triglyceride levels, smoking status, and body mass index (BMI). BDNF plasma levels were compared between cases and controls and mild and severe psoriasis using *t*-tests. A multivariate general linear model was also performed, with BDNF as the dependent variable and the aforementioned variables as predictors. Using this model, BDNF values are reported as estimated marginal means (95%CI), which adjusts the mean values of the other variables in the model. Significance was considered to be P<0.05.

### Results

**Figure 1:**
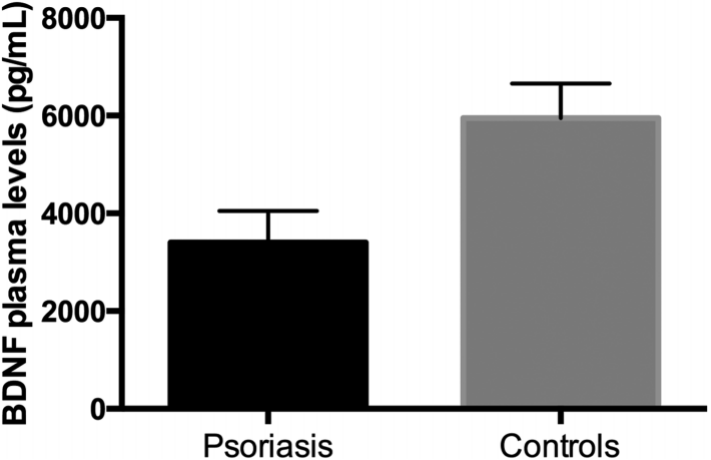
Brain-derived neurotrophic factor (BDNF) plasma levels in psoriasis patients (n=94) and controls (n=307). Data are reported as mean plasma BDNF and 95%CI upper limit. P≤0.01, psoriasis patients compared to controls (*t*-test).

Although similar in age and gender, psoriasis patients had higher BMI, SBP, HDLc, LDLc, and triglyceride levels compared with controls (Table 1). Additionally, BDNF plasma levels were significantly different between groups (P<0.01), being lower in psoriasis patients (Figure 1). The multivariate model (with all variables in Table 1 introduced as predictors) showed that BDNF levels remained significantly lower in psoriasis patients (P=0.01) (estimated marginal means of 3922 pg/mL; 95%CI=2660-5135) compared with controls (5788 pg/mL; 95%CI=5185-6442).

**Table:**
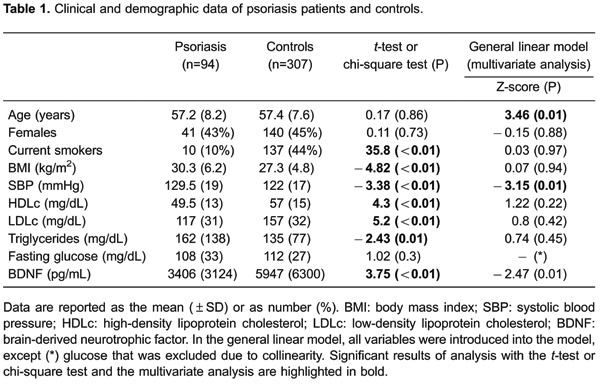

We detected no difference in BDNF levels between mild (M=3649; SD=3653) and severe (M=3280, SD=2837) psoriasis cases (t=0.53; P=0.59).

### Discussion

In this cross-sectional study, we examined 401 participants (94 with psoriasis), and found significantly lower BDNF levels in patients than controls. Our study strengths are the large sample number and exclusion of participants with depression (as BDNF levels are decreased in depression). Although different methods were used to exclude depression in cases and controls, both are similar in diagnosing depression. Indeed, PHQ-9≥10 has good specificity for excluding depression (12), whilst CIS-R is commonly used in cohort studies to diagnose mental illnesses (11).

Only Raap et al. (14) have previously examined BDNF levels in psoriasis, although their aim was to explore BDNF levels in atopic dermatitis, and psoriasis was included as a second control group (in addition to non-atopic subjects) without further characterization. Therefore, our results show for the first time that BDNF, a neurotrophin with a critical role in mental disorders and neuroplasticity, is also involved in psoriasis. It should be noted that we measured BDNF levels in blood and not the central nervous system, although BDNF crosses the blood-brain barrier by an active transport system (15).

Psychological stress is a possible factor linking decreased BDNF levels with psoriasis, as psoriasis patients experience increased distress and a lower quality of life compared with other diseases (16). Psychological stress activates the hypothalamic-pituitary-adrenal and sympathetic-adrenal-medullary axes, thereby increasing expression of cortisol and neuroinflammatory cytokines, and decreasing BDNF levels (7). BDNF has other skin-related functions, and induces apoptosis in normal, but not a psoriatic transit-amplifying sub-population of basal keratinocytes (17). Recently, a case-control study in 345 psoriasis cases and 315 controls showed a combined effect for the rs6265 GG genotype and higher BMI, with increased multivariate adjusted risk of psoriasis (OR=3.19; 95%CI=1.37-7.45) and psoriasis severity (OR=1.25; 95%CI=1.10-1.40) (18). It is not clear how these effects combine in psoriasis patients.

We observed no statistical difference between BDNF plasma levels in mild and severe psoriasis cases, highlighting the need for dermatologists to pay attention to psychological aspects of the disease, even in its milder forms. As our definition of severity was based mainly on the use of systemic immunosuppressants, it may highlight the chronic inflammatory nature of the illness, and despite adequate treatment, BDNF levels remain decreased. Indeed, immunosuppressant treatment is based on anti-tumor necrosis factor (TNF)-alpha therapy, as increased TNF-alpha activity is observed in psoriasis (16), and interestingly *in vitro* TNF-alpha stimulates BDNF expression in primary astrocytes (19). This may reflect the “double-edged blade” properties of TNF-alpha, in which its overexpression induces opposite effects i.e., down-regulates BDNF expression, and may also explain why severe psoriasis cases, in which systemic anti-TNF-alpha therapy is used, have similar BDNF levels as mild cases.

Another interesting finding from our study is increased BMI, SBP, and dyslipidemia in psoriasis, which reflects the concept of psoriasis being not only a skin, but also a systemic inflammatory disease associated with increased cardiovascular risk (16).

Our analysis has some limitations. The most important is that although BDNF affects several stages of psoriasis pathophysiology, low BDNF levels in psoriasis patients are only one piece of the puzzle. We did not identify higher stress levels in psoriasis patients compared with controls, but this may be a consequence of the specific questionnaire used in our study. There are other approaches for examining stress, with possibly different results, as observed in previous published studies (20). Another limitation is that we did not determine gamma-glutamyl transferase blood levels in the CALIPSO study. Therefore, we were unable to use gamma-glutamyl transferase as a proxy of alcohol abuse and a brain lesion marker, which in turn, may impact on BDNF blood levels. Also, as we performed a cross-sectional analysis, we cannot establish a cause and effect relationship between BDNF blood levels and psoriasis, and it is not possible to eliminate a possible “reverse causality” phenomenon.

Finally, no other measures of stress (e.g., salivary cortisol levels) were obtained in either the ELSA-Brasil or CALIPSO study, and we were unable to measure the impact of stress-related variables on our results. Nonetheless, our cases and controls did not have major depression as a possible confounder.

Our study further strengthens the concept of a connection between the brain and the skin in psoriasis, and shows decreased BDNF plasma levels in this disease. Future studies should determine if BDNF levels in psoriasis are associated with specific treatments and different disease stages, and also its role as a potential psoriasis biomarker.

## References

[1] Rampton DS (2011). The influence of stress on the development and severity
of immune-mediated diseases. J Rheumatol Suppl.

[2] Saraceno R, Kleyn CE, Terenghi G, Griffiths CE (2006). The role of neuropeptides in psoriasis. Br J Dermatol.

[3] Vasiadi M, Therianou A, Alysandratos KD, Katsarou-Katsari A, Petrakopoulou T, Theoharides A (2012). Serum neurotensin (NT) is increased in psoriasis and NT
induces vascular endothelial growth factor release from human mast
cells. Br J Dermatol.

[4] Paus R, Theoharides TC, Arck PC (2006). Neuroimmunoendocrine circuitry of the ‘brain-skin
connection’. Trends Immunol.

[5] Duman RS, Monteggia LM (2006). A neurotrophic model for stress-related mood
disorders. Biol Psychiatry.

[6] Brunoni AR, Lopes M, Fregni F (2008). A systematic review and meta-analysis of clinical
studies on major depression and BDNF levels: implications for the role of
neuroplasticity in depression. Int J Neuropsychopharmacol.

[7] Bath KG, Schilit A, Lee FS (2013). Stress effects on BDNF expression: effects of age, sex,
and form of stress. Neuroscience.

[8] Fuchikami M, Yamamoto S, Morinobu S, Takei S, Yamawaki S (2010). Epigenetic regulation of BDNF gene in response to
stress. Psychiatry Investig.

[9] Staniak HL, Bittencourt MS, de Souza Santos I, Sharovsky R, Sabbag C, Goulart AC (2014). Association between psoriasis and coronary calcium
score. Atherosclerosis.

[10] Brunoni AR, Santos IS, Sabbag C, Lotufo PA, Bensenor IM (2014). Psoriasis severity and hypothalamic-pituitary-adrenal
axis function: results from the CALIPSO study. Braz J Med Biol Res.

[11] Aquino EM, Barreto SM, Bensenor IM, Carvalho MS, Chor D, Duncan BB (2012). Brazilian Longitudinal Study of Adult Health
(ELSA-Brasil): objectives and design. Am J Epidemiol.

[12] de Lima Osorio F, Vilela Mendes A, Crippa JA, Loureiro SR (2009). Study of the discriminative validity of the PHQ-9 and
PHQ-2 in a sample of Brazilian women in the context of primary health
care. Perspect Psychiatr Care.

[13] Nunes MA, Alves MGM, Chor D, Schimdt MI, Duncan BB (2011). Adaptação transcultural do CIS-R (clinical interview
schedule - revised version) para o Português no estudo longitudinal de saúde do
adulto (ELSA). Rev HCPA.

[14] Raap U, Werfel T, Goltz C, Deneka N, Langer K, Bruder M (2006). Circulating levels of brain-derived neurotrophic factor
correlate with disease severity in the intrinsic type of atopic
dermatitis. Allergy.

[15] Pan W, Banks WA, Fasold MB, Bluth J, Kastin AJ (1998). Transport of brain-derived neurotrophic factor across
the blood-brain barrier. Neuropharmacology.

[16] Nestle FO, Kaplan DH, Barker J (2009). Psoriasis. N Engl J Med.

[17] Truzzi F, Marconi A, Atzei P, Panza MC, Lotti R, Dallaglio K (2011). p75 neurotrophin receptor mediates apoptosis in
transit-amplifying cells and its overexpression restores cell death in psoriatic
keratinocytes. Cell Death Differ.

[18] Quan C, Zhu KJ, Zhang C, Liu Z, Liu H, Zhu CY (2014). Combined effects of the BDNF rs6265 (Val66Met)
polymorphism and environment risk factors on psoriasis vulgaris. Mol Biol Rep.

[19] Saha RN, Liu X, Pahan K (2006). Up-regulation of BDNF in astrocytes by TNF-alpha: a case
for the neuroprotective role of cytokine. J Neuroimmune Pharmacol.

[20] Consoli SM, Rolhion S, Martin C, Ruel K, Cambazard F, Pellet J (2006). Low levels of emotional awareness predict a better
response to dermatological treatment in patients with psoriasis. Dermatology.

